# Effects of Fermented Grape Seed in Breeding-Pigeon Diets on Growth Performance, Immune Responses, and Oxidative Status of Offspring Squabs

**DOI:** 10.3390/ani16172692

**Published:** 2026-08-29

**Authors:** Honglei Sun, Huiguo Yang, Haiying Li, Xiaobin Li, Yuanhao Li, Yafei Liang, Jie Ren, Jiajia Liu, Xiaoyu Zhao, Yingping Wu, Aikemu Mamaitijiang

**Affiliations:** 1 College of Animal Science, Xinjiang Agricultural University, Urumqi 830052, China; sunhonglei0805@163.com (H.S.);; 2 Institute of Animal Husbandry, Xinjiang Academy of Animal Husbandry, Urumqi 830011, China; 3 Moyu Blue Sea Pigeon Industry Co., Ltd., Hetian 848100, China

**Keywords:** parental nutrition, pigeon production, antioxidant capacity, inflammatory response, serum biochemistry, meat quality, dose-response

## Abstract

This study evaluated the effects of graded fermented grape seed (FGS) supplementation in breeding-pigeon diets on the growth performance, immune responses, and redox status of squabs. Forty-eight healthy pairs of Gray King breeding pigeons were randomly assigned to four treatments, with 12 cages per treatment. Breeding pigeons received the experimental diets beginning 7 d before the expected hatch date. The basal diet was supplemented with 0, 10, 20, or 30 kg FGS per 1000 kg of diet (CON, FGS10, FGS20, and FGS30, respectively), and each pair reared three squabs until 28 d of age. Growth performance, carcass traits, meat quality, serum biochemical parameters, immunoglobulins, inflammatory cytokines, and serum and hepatic redox indices were evaluated. FGS supplementation affected body weight at 14 d of age and average daily gain (ADG) from 14 to 21 d and from 14 to 28 d of age (*p* < 0.05), but did not affect body weight at 28 d of age. FGS30 reduced ADG from 14 to 21 and 14 to 28 d and increased relative carcass yields, immunoglobulin A, G, and M levels, interleukin-10, tumor necrosis factor-α, aspartate aminotransferase, low-density lipoprotein cholesterol, and serum and hepatic malondialdehyde levels, while decreasing hepatic superoxide dismutase and glutathione peroxidase activities. FGS20 and FGS30 increased breast muscle L* values and press loss. FGS20 showed numerical advantages in several production traits, but increased breast muscle press loss and did not improve hepatic redox status. Overall, FGS supplementation in breeding-pigeon diets resulted in stage-specific and dose-related responses. FGS30 may exceed the appropriate inclusion range. Based on the present data, none of the tested supplementation levels can be considered optimal, and the range of 10–20 kg FGS per 1000 kg basal diet requires further validation.

## Simple Summary

Fermented grape seed (FGS) is produced from grape-processing by-products and contains a variety of nutrients and bioactive compounds, indicating its potential value as a feed ingredient. During early growth, squabs depend primarily on parental feeding; therefore, the composition of the parental diet may influence their growth and physiological status through parental nutrient transfer. In the present study, FGS was added to breeding-pigeon diets to evaluate its effects on growth performance, carcass traits, meat quality, immune responses, and redox status in squabs. The results suggest that dietary supplementation with 30 kg FGS per 1000 kg of diet may exceed an appropriate inclusion level, as evidenced by increases in inflammation-related indices, lipid peroxidation markers, and several serum metabolic parameters. FGS20 showed numerical advantages in several production traits but also increased breast muscle press loss and did not improve hepatic redox status. Therefore, none of the tested supplementation levels can be considered optimal or recommended, and the range of 10–20 kg FGS per 1000 kg of diet requires further validation. These findings provide information to guide the rational use of grape-processing by-products in meat pigeon diets.

## 1. Introduction

Pigeon production is a specialized poultry sector that contributes to the supply of animal protein for human consumption. In commercial pigeon production, the growth and health of squabs are important determinants of production efficiency, product quality, and economic returns. Because squabs depend almost entirely on parental feeding during the early post-hatch period, nutritional management of breeding pigeons is of particular practical importance. Squabs are typical altricial birds that cannot feed independently after hatching and initially obtain nutrients mainly from crop milk secreted by their parents and from regurgitated, crop-softened feed. Crop milk is rich in protein and lipids and contains immune-related components and a characteristic microbiota; its composition is influenced by the nutritional status of breeding pigeons [1,2,3,4,5]. Consequently, manipulating diets for breeding pigeons can affect breeders themselves and indirectly alter squab growth, immune development, and redox homeostasis via parental nutrient transfer. Previous studies have shown that fatty acids, protein, riboflavin, zinc, polyphenols, and fermented feeds in breeding-pigeon diets can affect squab growth, antioxidant capacity, immune indices, or intestinal function [2,3,4,5,6,7,8,9,10,11].

Grape-processing by-products typically include grape skins, seeds, and residual pulp. Grape seeds contain dietary fiber, residual protein and lipids, minerals, and various bioactive compounds, particularly proanthocyanidins, catechins, and other polyphenols. These components may provide both nutritional value and functional benefits, including antioxidant activity and potential modulation of oxidative and metabolic processes. In broilers, grape-seed extract and grape proanthocyanidins can modulate lipid metabolism, antibody responses, and redox-related indices; however, their effects depend on dose, product form, basal-diet composition, and the physiological status of the animal [12,13,14,15,16,17,18]. Low doses of polyphenols may exert free-radical-scavenging and signaling effects, whereas higher doses or complex plant materials may also exert pro-oxidant effects or limit nutrient utilization [19,20]. Therefore, an increase in a single antioxidant enzyme activity or immunoglobulin concentration is insufficient to demonstrate an overall physiological benefit of a higher inclusion level.

Overall, previous studies suggest that grape-derived products may exert beneficial effects on growth performance and antioxidant-related responses in poultry, but the magnitude and direction of these effects vary among products and inclusion levels. Most available evidence has been obtained from broilers or from isolated grape-derived extracts, whereas evidence from breeding pigeons and their indirectly exposed squabs remains limited. Moreover, the integrated effects of different inclusion levels on growth, immunity, inflammation, and redox status have not been systematically evaluated in squabs. Microbial fermentation can degrade some cell-wall components and antinutritional factors and alter the availability of polyphenols, oligopeptides, and microbial metabolites in plant by-products. Fermented grape seed (FGS) has also been reported to improve growth performance and antioxidant responses in broilers [12]. In that study, broilers were fed a control diet or diets supplemented with 5 g/kg GS or FGS for 42 days, with 0.15 g/kg butylated hydroxyanisole included as a synthetic antioxidant reference. Dietary FGS increased body weight, average daily gain, and serum catalase activity compared with the control group [12], thereby providing a basis for evaluating different FGS inclusion levels in the present study. However, few studies have evaluated FGS in a production system in which breeding pigeons consume the experimental diets and squabs are indirectly exposed through parental feeding. In particular, the integrated effects of different inclusion levels on humoral immunity, inflammatory responses, and serum and hepatic redox status in squabs remain unclear.

Because the FGS used in the present study differed from products used in previous studies in terms of source and fermentation process, its composition and biological effects may also differ. Accordingly, the inclusion levels in the present study were selected primarily on the basis of the supplier-recommended dosage for this product, whereas previously reported inclusion levels were used only as references rather than adopted directly. FGS was used as an additional dietary supplement rather than as a replacement for a specific basal-diet ingredient; consequently, the experimental diets were not formulated to be isonitrogenous or isoenergetic. In the present study, breeding-pigeon diets were supplemented with 0, 10, 20, or 30 kg FGS per 1000 kg basal diet, and squab growth performance, immune and inflammatory responses, and serum and hepatic redox status were primarily evaluated. Body measurements, carcass traits, meat quality, and serum biochemical indices were included as supplementary endpoints. We hypothesized that the effects of FGS on squab production-related traits and physiological status would depend on the inclusion level and that higher inclusion levels might be accompanied by changes in redox homeostasis and metabolic responses.

## 2. Materials and Methods

### 2.1. Ethical Statement

All animal husbandry, blood collection, slaughter, and tissue-sampling procedures were conducted in accordance with animal welfare requirements and were reviewed and approved by the Animal Ethics Committee of Xinjiang Agricultural University (approval no. 202602; approval date: 23 March 2026).

### 2.2. Fermented Grape Seed Product and Experimental Diets

Fermented grape seed (FGS) was supplied by Zhanjiang Boshan Jiuqian Biotechnology Co., Ltd. (Zhanjiang, Guangdong, China). The product was produced from fresh, non-defatted grape pomace, a grape-processing residue originating from Xinjiang, by solid-state fermentation with *Lactiplantibacillus plantarum*, *Saccharomyces cerevisiae*, and *Bacillus subtilis* at 28–32 °C for 5 d. After fermentation, the product was dried in a forced-air oven at 40 °C, ground to 20–40 mesh, thoroughly mixed, sealed, and stored under moisture-proof conditions. Additional compositional analyses were performed on the same batch of FGS used in the feeding trial. The analyzed chemical composition and selected physicochemical characteristics of FGS are presented in Table 1.

The basal diet was formulated using corn, peas, soybean meal, wheat middlings, yeast powder, and other ingredients; its ingredient composition and calculated nutrient content are shown in Table 2. The analyzed dry matter content of the basal pelleted diet was 92.18%. On a dry-matter basis, the basal diet contained 17.06% crude protein, 4.83% ether extract, 16.26% neutral detergent fiber, 5.35% acid detergent fiber, and 10.26% ash.

CON, FGS10, FGS20, and FGS30 denote supplementation with 0, 10, 20, and 30 kg FGS, respectively, per 1000 kg basal diet. Because FGS was added as an additional supplement rather than used to replace an equivalent amount of a basal-diet ingredient, the resulting total batch weights were 1000, 1010, 1020, and 1030 kg, respectively. Based on the analyzed FGS dry matter content of 94.17%, the FGS10, FGS20, and FGS30 treatments supplied approximately 9.42, 18.83, and 28.25 kg of FGS dry matter, respectively. FGS was first premixed with a small amount of the basal diet and then thoroughly mixed with the remaining basal diet. Identical mixing and storage conditions were used for all treatments. The four final mixed diets were not analyzed separately; therefore, their estimated proximate compositions, derived from the analyzed compositions of the basal diet and FGS and their actual inclusion levels, are presented in Table 2.

For the estimated proximate composition, CON, FGS10, FGS20, and FGS30 represent the addition of 0, 10, 20, and 30 kg of FGS, respectively, per 1000 kg of basal diet. Values were estimated from the analyzed dry matter contents and dry-matter nutrient compositions of the basal pelleted diet and FGS, weighted according to the actual inclusion levels. The four final mixed diets were not analyzed separately; therefore, the reported values are calculated estimates rather than directly measured values.

### 2.3. Animals and Experimental Design

The experiment was conducted from 16 April to 21 May 2026 at Lanhai Pigeon Farm, Moyu County, Hotan Prefecture, Xinjiang Uygur Autonomous Region, China. A completely randomized design was used. Forty-eight cages of clinically healthy Gray King breeding pigeons with similar laying dates and expected hatching dates were selected, with each cage containing one male and one female. The cage was the experimental unit, and cages were randomly assigned to CON, FGS10, FGS20, or FGS30, with 12 replicates per treatment. Breeding pigeons began receiving the corresponding experimental diets 7 d before the expected hatch. After hatching, each pair naturally reared three squabs to 28 d of age. All treatments were housed in the same pigeon house under identical husbandry, hygiene, and environmental management conditions. The photoperiod was 16 h/d, the house temperature was 15 ± 5 °C, and breeding pigeons had ad libitum access to feed and water.

### 2.4. Measurements

#### 2.4.1. Growth Performance

At 7, 14, 21, and 28 d of age, feed consumption was recorded for a continuous 24 h period on a cage basis and calculated as the amount of feed offered minus the amount remaining. This measurement represented the combined feed consumption of one pair of breeding pigeons and three squabs within a family cage and was expressed as g/cage/24 h; it was not interpreted as feed intake per squab or as a conventional feed conversion ratio. At 14, 21, and 28 d of age, body weight (g), body oblique length (cm), keel length (cm), shank circumference (cm), shank length (mm), chest width (mm), and chest depth (mm) were measured individually in all three squabs per cage. These body measurements were used as complementary indicators of squab growth and body development. Body oblique length and keel length reflected longitudinal and skeletal development, whereas chest width, chest depth, shank length, and shank circumference provided additional information on body conformation and breast and limb development. Body measurements were obtained according to Terminology and Measurement Methods for Poultry Production Performance (NY/T 823-2020) [21]. Body oblique length was measured along the body surface from the shoulder joint to the ipsilateral ischial tuberosity; keel length was measured from the anterior to the posterior end of the keel; chest depth was measured from the first thoracic vertebra to the anterior edge of the keel; chest width was measured between the two shoulder joints; shank length was measured from the proximal metatarsal joint to the point between the third and fourth toes; and shank circumference was measured at the midpoint of the shank. Body oblique length, keel length, and shank circumference were measured using a non-stretchable tape, whereas chest depth, chest width, and shank length were measured using a vernier caliper. All measurements were performed by the same trained operator at similar times of day using the same instruments. The mean of the three squabs in each cage was treated as one experimental replicate. Average daily gain (ADG) from 14 to 21, 21 to 28, and 14 to 28 d was calculated as the change in cage-mean body weight over the corresponding interval divided by the number of days.

#### 2.4.2. Serum Biochemical, Redox, Immune, and Inflammatory Indices

At 28 d of age, after an 8 h fasting period with free access to water, one squab was randomly selected from each cage, and blood was collected from the wing vein. Blood was allowed to clot at 20–25 °C for 30 min and centrifuged at 3000 rpm for 10 min at 4 °C. Serum was separated and stored at −80 °C. Serum biochemical indices included total protein (TP; A045-1), albumin (ALB; A028-2), alanine aminotransferase (ALT; C009-2), aspartate aminotransferase (AST; C010-2), triglycerides (TG; A110-1), total cholesterol (TC; A111-1), high-density lipoprotein cholesterol (HDL-C; A112-1), and low-density lipoprotein cholesterol (LDL-C; A113-1). Serum redox indices included total superoxide dismutase (T-SOD; A001-1), malondialdehyde (MDA; A003-1), glutathione peroxidase (GSH-Px; A005-1), catalase (CAT; A007-1), and total antioxidant capacity (T-AOC; A015-2). Kits for the serum biochemical and redox indices were purchased from Nanjing Jiancheng Bioengineering Institute (Nanjing, China) and used according to the manufacturer’s instructions. Serum IgA, IgG, IgM, IL-1β, IL-6, IL-10, and TNF-α were measured by the Testing Department of Nanjing Jiancheng Bioengineering Institute using pigeon-specific competitive ELISA kits (lot no. 202606). A 50 μL aliquot of each sample was used; samples for IL-1β, IL-6, and TNF-α were diluted twofold before analysis, whereas the other indices were measured without dilution. All procedures followed the corresponding kit instructions. Absorbance was read at 450 nm using an HBS-1096A microplate reader (Nanjing Detie Laboratory Equipment Co., Ltd., Nanjing, China), and concentrations were calculated from the standard curves.

#### 2.4.3. Hepatic Indices

After slaughter, tissue was collected from the central region of the left hepatic lobe, gently rinsed with precooled phosphate buffer, rapidly aliquoted, and stored at −80 °C. Homogenates were prepared on ice at a tissue-to-buffer ratio of 1:10 (g:mL) and centrifuged at 2500 rpm for 10 min. The resulting supernatant was collected. Protein concentration was determined using a Coomassie Brilliant Blue protein assay kit (A045-2; Nanjing Jiancheng Bioengineering Institute). SOD (A001-1), MDA (A003-1), GSH-Px (A005-1), and T-AOC (A015-2) were measured, and the results were normalized to protein concentration.

#### 2.4.4. Carcass Traits and Meat Quality

According to NY/T 823-2020 [21], squabs were fasted for 8 h with free access to water before slaughter at 28 d of age. One squab was randomly selected from each cage, yielding 12 squabs per treatment. Preslaughter live weight was recorded after fasting. Squabs were euthanized by carotid artery exsanguination and completely exsanguinated before defeathering. Carcass weight, semi-eviscerated weight, and eviscerated weight were determined according to NY/T 823-2020. Semi-eviscerated weight was defined as the carcass weight after removal of the digestive tract and its contents, with the head, feet, and edible giblets retained. The left breast and thigh muscles were dissected and weighed. Abdominal fat and the heart, liver, spleen, kidneys, proventriculus, gizzard, and bursa of Fabricius were also collected and weighed. Carcass yield, semi-eviscerated yield, and eviscerated yield were calculated as the corresponding weight divided by preslaughter live weight × 100%. Organ indices were calculated as organ weight divided by preslaughter live weight × 100%. The relative weights of the left breast and thigh muscles were calculated as the corresponding muscle weight divided by eviscerated weight × 100%.

At 45 min postmortem, pH values of the breast and thigh muscles were measured using a portable pH-Star meter (Matthäus GmbH & Co. KG, Eckelsheim, Germany), and L*, a*, and b* values were measured using an NR110 portable colorimeter (Shenzhen 3nh Technology Co., Ltd., Shenzhen, China; 4 mm aperture; d/8° optical geometry). The instruments were calibrated according to the manufacturers’ instructions. Measurements were obtained at three sites within the same anatomical region while avoiding visible fat, fascia, blood spots, and damaged areas; the mean of the three measurements was used. Press loss was calculated as (weight before pressing − weight after pressing)/weight before pressing × 100%. For shear-force measurement, visible fat, tendons, and connective tissue were removed from breast and thigh muscle samples. Samples were placed separately in heat-resistant sealed bags and heated in an 80 °C thermostatic water bath (DK-S24) until the core temperature reached 70 °C. The samples were removed, cooled to room temperature, blotted dry, and cut into uniform strips parallel to the muscle fibers. Shear force was measured perpendicular to the muscle fibers using a C-LM3B digital meat tenderness meter (Beijing Tianxiang Feiyu Instrument and Equipment Co., Ltd., Beijing, China). Three valid measurements were obtained per sample, and their mean was recorded in N.

### 2.5. Statistical Analysis

Data were analyzed using IBM SPSS Statistics 27.0 (IBM Corp., Armonk, NY, USA), with the cage as the experimental unit. Repeated measurements of 24 h feed consumption per family cage and cage-mean body weight were analyzed using repeated-measures models. The model was Yijk = μ + Ti + Aj + (T × A)ij + Ck(i) + εijk, where Yijk is the observed value, μ is the overall mean, Ti is the fixed effect of dietary treatment (CON, FGS10, FGS20, or FGS30), Aj is the fixed effect of age, (T × A)ij is the treatment × age interaction, Ck(i) represents the cage nested within treatment as the repeated-measures subject, and εijk is the residual error. Age was treated as the repeated factor. Sphericity was assessed using Mauchly’s test; when the assumption was violated, the Greenhouse–Geisser correction was applied. Body weight and body measurements at individual ages, ADG, carcass traits, organ indices, meat quality, and serum and hepatic indices were analyzed separately using a one-way model. The model was Yij = μ + Ti + εij, where Yij is the observed value, μ is the overall mean, Ti is the fixed effect of dietary treatment, and εij is the residual error. Homogeneity of variance was assessed using Levene’s test. When Levene’s test yielded *p* < 0.05, Welch’s robust test was applied, followed by Games–Howell comparisons when the overall treatment effect was significant. For variables meeting the homogeneity assumption, Duncan’s multiple-range test was used when the overall treatment effect was significant. Prespecified linear and quadratic orthogonal polynomial contrasts were conducted using the equally spaced FGS supplementation levels of 0, 10, 20, and 30 kg per 1000 kg basal diet. Results are presented as means with the pooled standard error of the mean (pooled SEM). Differences were considered significant at *p* < 0.05, and 0.05 ≤ *p* < 0.10 was considered a trend.

## 3. Results

### 3.1. Growth Performance and Feed Consumption

Repeated-measures analysis of body weight showed significant effects of age and the treatment × age interaction (*p* < 0.001 and *p* = 0.013, respectively; the interaction was corrected using the Greenhouse–Geisser method), whereas the overall treatment effect was not significant (*p* = 0.239; Table 3). Age-specific analyses indicated that FGS supplementation significantly affected body weight at 14 d and ADG from 14 to 21 and 14 to 28 d (*p* < 0.05). Compared with CON, body weight at 14 d was significantly higher in FGS10, FGS20, and FGS30 (*p* < 0.05) and increased linearly (*p* = 0.002). ADG from 14 to 21 d was significantly lower in FGS30 than in the other groups (*p* < 0.05) and decreased linearly (*p* < 0.001). ADG from 14 to 28 d was significantly lower in FGS30 than in CON and FGS20 (*p* < 0.05), with FGS10 showing an intermediate value. Body weight at 21 and 28 d and ADG from 21 to 28 d did not differ among treatments (*p* > 0.05). FGS supplementation did not significantly affect 24 h feed consumption per family cage at 7, 14, 21, or 28 d (*p* > 0.05). Repeated-measures analysis of feed consumption showed a significant age effect (*p* < 0.001), whereas the overall treatment effect and treatment × age interaction were not significant (*p* = 0.504 and *p* = 0.964, respectively).

**Figure 1:**
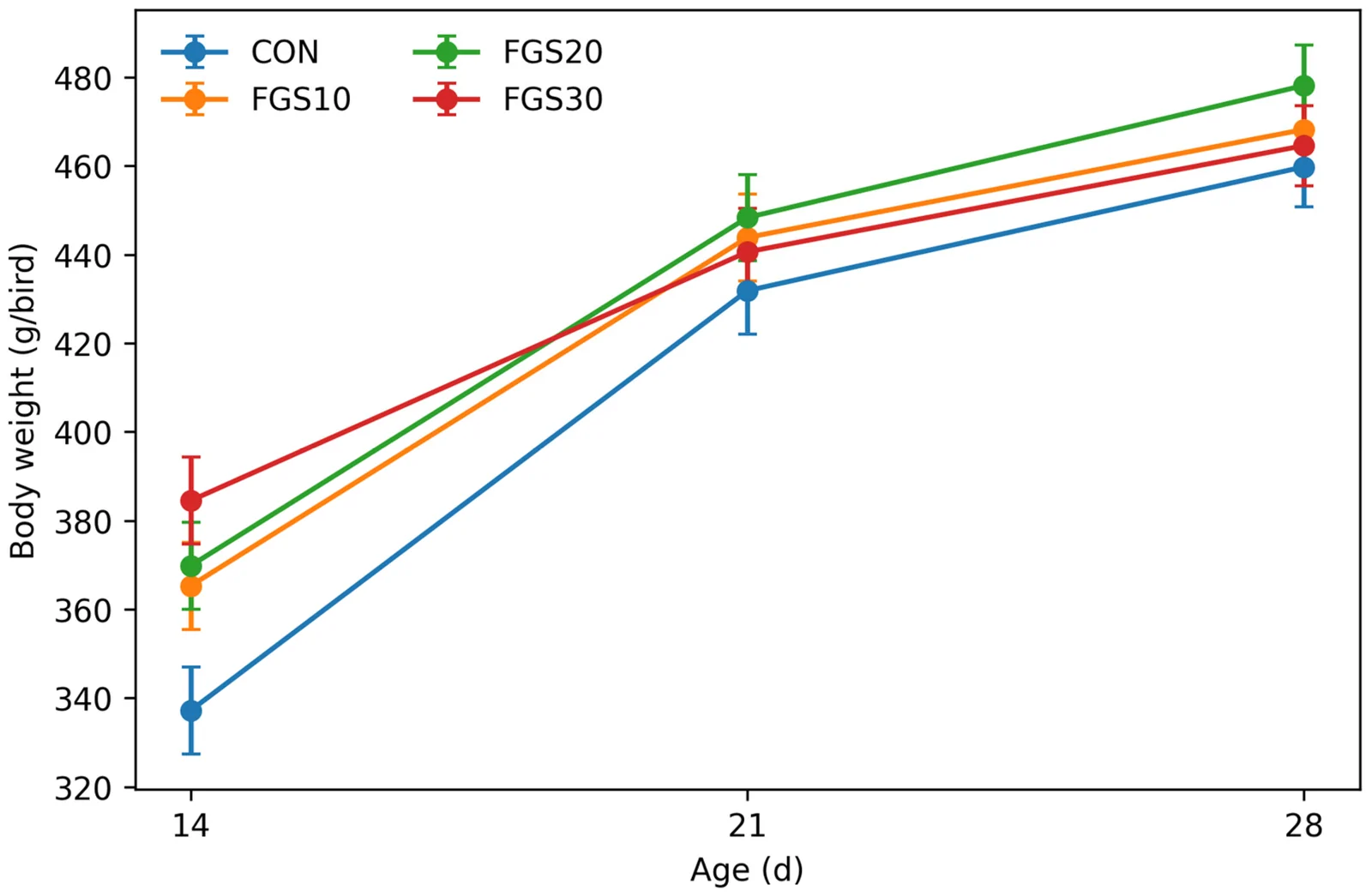
Changes in body weight of squabs from 14 to 28 d of age in response to graded FGS supplementation. Values are means ± pooled SEM (*n* = 12 cages per treatment; each cage value is the mean of three squabs).

The significant treatment × age interaction for body weight indicated that the pattern of body-weight change across ages differed among dietary treatments; accordingly, treatment differences were examined at each age. For 24 h feed consumption per family cage, the significant age effect, together with nonsignificant treatment and treatment × age effects, indicated that feed consumption changed with age, but its temporal pattern did not differ among dietary treatments. The temporal changes in squab body weight across the four dietary treatment groups are illustrated in Figure 1.

### 3.2. Serum and Hepatic Redox Status

FGS supplementation significantly affected serum MDA and GSH-Px, as well as hepatic SOD, MDA, and GSH-Px (*p* < 0.05; Table 4). Serum MDA increased linearly with supplementation level and was significantly higher in FGS30 than in CON and FGS10. Serum GSH-Px was significantly higher in FGS30 than in CON and FGS20. The overall treatment effects on serum T-SOD, CAT, and T-AOC were not significant (*p* > 0.05); however, the quadratic contrast indicated a quadratic response for serum T-AOC (*p* = 0.044). Hepatic MDA was significantly higher, and hepatic GSH-Px was significantly lower in all three FGS groups than in CON. Hepatic SOD was significantly lower in FGS10 and FGS30 than in CON, with FGS20 being intermediate. The overall treatment effect on hepatic T-AOC was not significant (*p* = 0.061), although the quadratic contrast indicated a quadratic response (*p* = 0.035). These significant quadratic contrasts were interpreted as nonlinear dose-response patterns rather than as evidence of significant overall treatment effects.

### 3.3. Immunoglobulins and Inflammatory Cytokines

FGS supplementation significantly affected serum IgA, IgG, IgM, IL-10, and TNF-α in squabs (*p* < 0.001), and all these indices increased linearly with supplementation level (*p* < 0.001; Table 5). IgA, IgM, IL-10, and TNF-α were significantly higher in FGS30 than in the other three groups. IgG was significantly higher in FGS30 than in CON and FGS20, with FGS10 being intermediate (*p* < 0.05). The overall treatment effect on IL-6 tended to be significant (*p* = 0.055), and IL-6 increased linearly with supplementation level (*p* = 0.014). The variance was heterogeneous for IL-1β, and Welch’s test indicated no significant difference among groups (*p* = 0.122).

### 3.4. Serum Biochemical and Lipid-Metabolism Indices

FGS supplementation significantly affected serum AST and LDL-C in 28-day-old squabs (*p* < 0.05; Table 6). AST was significantly higher in FGS30 than in CON, FGS10, and FGS20 (*p* = 0.003) and showed both linear and quadratic responses (*p* = 0.002 and *p* = 0.048, respectively). LDL-C was significantly higher in FGS30 than in CON, with FGS10 and FGS20 being intermediate (*p* = 0.013), and increased linearly with supplementation level (*p* = 0.003). TP, ALB, ALT, TG, TC, and HDL-C showed no significant treatment effects (*p* > 0.05), although ALB tended to be affected by treatment (*p* = 0.058). The variance was heterogeneous for TG, and Welch’s test indicated no significant treatment effect (*p* = 0.793).

### 3.5. Carcass Traits and Organ Indices

FGS supplementation significantly affected carcass yield, semi-eviscerated yield, and eviscerated yield in 28-day-old squabs (*p* < 0.05; Table 7). All three carcass-yield indices were significantly higher in FGS30 than in CON, FGS10, and FGS20 and increased linearly with supplementation level (*p* < 0.05); carcass yield also showed a quadratic response (*p* = 0.005). Preslaughter live weight, carcass weight, semi-eviscerated weight, eviscerated weight, breast muscle weight, thigh muscle weight, abdominal fat weight, and abdominal fat percentage did not differ significantly among groups (*p* > 0.05). FGS supplementation did not significantly affect the relative weights of the breast or thigh muscles (*p* > 0.05).

FGS supplementation did not significantly affect the absolute weights or relative indices of the heart, liver, spleen, kidney, proventriculus, gizzard, or bursa of Fabricius (*p* > 0.05; Supplementary Table S2). Liver weight decreased linearly with increasing supplementation level (*p* = 0.028), and the liver index tended to decrease linearly (*p* = 0.051), although neither overall treatment effect was significant.

### 3.6. Meat Quality

FGS supplementation significantly affected breast muscle L*, breast muscle b*, thigh muscle a*, thigh muscle b*, and breast muscle press loss in squabs (*p* < 0.05; Table 8). Breast muscle L* and press loss were significantly higher in FGS20 and FGS30 than in CON and FGS10 and increased linearly with supplementation level (*p* < 0.001). Breast muscle b* was significantly higher in FGS20 than in CON and FGS10, with FGS30 being intermediate. Thigh muscle a* was significantly higher in FGS20 than in CON, with FGS10 and FGS30 being intermediate. Thigh muscle b* was higher in all three FGS groups than in CON and was highest in FGS20.

The overall treatment effect on breast muscle pH was not significant (Welch *p* = 0.112), but pH decreased linearly with supplementation level (*p* = 0.013). The overall treatment effect on thigh muscle press loss showed a trend toward significance (Welch *p* = 0.068), and press loss increased linearly (*p* = 0.019). Thigh muscle pH, breast muscle a*, thigh muscle L*, and breast and thigh muscle shear force did not differ significantly among groups (*p* > 0.05).

## 4. Discussion

### 4.1. Overall Dose–Response Effects of Parental FGS Supplementation on Squab Growth and Physiological Responses

The present study showed stage- and dose-dependent responses to parental FGS supplementation rather than progressively greater benefits as the inclusion level increased. FGS30 was associated with reduced growth during specific periods and a less favorable inflammatory, metabolic, and redox profile, whereas the numerical production advantages observed with FGS20 were not accompanied by significant improvements in final body weight or hepatic redox status.

During early growth, squabs obtain nutrients mainly from crop milk and regurgitated, softened feed provided by their parents. Thus, dietary FGS may influence squab growth through nutrient transfer during parental feeding [1,2,3,4,5]. In the present study, body weight at 14 d was significantly higher in all three FGS groups than in CON, and several body measurements at 14 d also varied with supplementation level, indicating that dietary intervention in breeding pigeons can alter early growth-related traits in squabs. However, body weight at 21 and 28 d did not differ significantly among groups, and ADG from 14 to 21 and 14 to 28 d was reduced in FGS30, indicating that the early body-weight differences did not result in a sustained final growth advantage.

The reduction in ADG at the highest FGS inclusion level was not accompanied by a significant decrease in measured 24 h feed consumption per family cage, suggesting that the lower growth rate cannot be explained simply by reduced feed consumption. Chemical characterization of the FGS product showed that it contained total phenols (20.25 mg GAE/g), total flavonoids (19.16 mg/g, expressed as rutin equivalents), and total acids (4.88 g/kg, expressed as lactic acid), with a pH of 4.2. Thus, increasing FGS inclusion also increased dietary exposure to these bioactive and fermentation-derived components. Although polyphenols may exert antioxidant and physiological regulatory effects at appropriate levels, their effects can be dose-dependent, and greater exposure does not necessarily result in greater biological benefit. In the present study, FGS30 was also associated with increased serum and hepatic MDA, TNF-α, AST, and LDL-C and reduced hepatic antioxidant enzyme activities. Taken together, these findings suggest that the reduced ADG at the highest inclusion level may reflect a less favorable balance between nutrient utilization and physiological responses at this inclusion level. However, a causal mechanism cannot be established from the present data.

FGS20 had the highest mean 28 d body weight and ADG from 21 to 28 d; its ADG from 14 to 28 d was statistically similar to that of CON, and several absolute carcass weights were numerically higher. However, these indices did not differ significantly from CON, and the present results do not identify a definitive optimal level. The absence of a sustained final body-weight advantage in the present study is consistent with the variable productive responses reported for grape-derived ingredients in poultry, which appear to depend on ingredient composition, inclusion rate, and physiological stage [22,23,24]. This pattern is also consistent with the stage-specific response observed here, in which early body weight increased without a significant improvement in final body weight. Therefore, the range of 10–20 kg FGS per 1000 kg basal diet requires further validation, whereas the responses observed at 30 kg/t suggest that increasing FGS inclusion beyond this range may not confer additional production benefits.

### 4.2. Humoral Immune Responses

The higher serum IgA, IgG, and IgM concentrations in FGS30 indicate enhanced humoral immune responses in squabs at the highest inclusion level. IgA, IgG, and IgM participate in mucosal defense, humoral immune effector functions, and primary immune responses, respectively; however, higher immunoglobulin concentrations measured at a single time point do not necessarily indicate improved disease resistance. Previous studies have shown that nutritional regulation in breeding pigeons can affect immune indices in squabs [6,8]. The present findings further indicate that the immune-related effects of FGS were dose-dependent.

The simultaneous increases in IL-10 and TNF-α in FGS30, together with the linear increase in IL-6, are consistent with concurrent anti-inflammatory counter-regulation and enhanced pro-inflammatory signaling. Because vaccine antibody titers, pathogen challenge, immune-organ histology, and cell-mediated immunity were not evaluated, the present findings are more appropriately described as enhanced immune-related responses accompanied by increased inflammatory signaling rather than as unequivocally beneficial immune enhancement or pathological inflammation. Previous broiler studies similarly indicate that grape-pomace interventions can modulate both humoral and inflammatory immune responses [25,26]. However, differences in intervention route and health status limit direct comparisons with the present breeding-pigeon model. Accordingly, the increased immunoglobulin concentrations observed here are better interpreted as evidence of immune modulation rather than improved disease resistance. Taken together, the simultaneous increases in immunoglobulins, TNF-α, and MDA in FGS30 indicate immune modulation accompanied by increased inflammatory signaling and lipid peroxidation, rather than an unequivocal improvement in immune status.

### 4.3. Redox Regulation and Metabolic Adaptation

Serum GSH-Px was increased in FGS30, but serum MDA was also significantly increased, whereas the overall treatment effects on T-SOD, CAT, and T-AOC were not significant. Although the concurrent increases in GSH-Px and MDA may appear contradictory, increased antioxidant enzyme activity can occur as a compensatory response to enhanced oxidative challenge and lipid peroxidation. Similar concurrent increases in MDA and antioxidant-enzyme activities have been reported in poultry under oxidative stress conditions [27]. Therefore, the increased serum GSH-Px in FGS30 should not be interpreted as evidence of improved overall antioxidant status, particularly in the presence of increased MDA. Fermentation may alter the chemical profile and biological activity of grape-derived materials. However, the present study did not compare the product before and after fermentation or characterize potentially undesirable fermentation-derived compounds; therefore, it is not possible to determine whether fermentation-related compositional changes contributed to the redox responses observed in FGS30. In addition, grape-seed polyphenols themselves may exhibit antioxidant or pro-oxidant effects depending on dose and surrounding conditions [19]. Further characterization of fermentation metabolites and polyphenol transformation products is therefore warranted.

Increases in serum MDA, AST, and TNF-α were less pronounced in FGS20 than in FGS30, indicating that these serum changes were less marked in FGS20. Previous poultry studies have generally reported antioxidant-related benefits of grape-derived products, including improvements in selected redox indices and reductions in lipid peroxidation, although the responses vary with inclusion level and experimental conditions [22,25,28,29]. In contrast, the concurrent increase in serum GSH-Px and MDA and the unfavorable hepatic redox responses observed in the present study indicate that FGS did not confer a consistent antioxidant benefit. This discrepancy suggests that product composition, inclusion level, intervention route, and animal physiological stage may substantially influence the redox effects of grape-derived materials.

Hepatic MDA increased and hepatic GSH-Px decreased in all three FGS groups, whereas hepatic SOD also decreased in FGS10 and FGS30, indicating that FGS did not improve hepatic redox status under the present supplementation conditions. The increases in AST and LDL-C in FGS30 may indicate a greater metabolic burden at the highest inclusion level. However, because AST is not liver-specific and hepatic histology was not evaluated, the increased AST should not be interpreted as direct evidence of liver injury. FGS20 also showed unfavorable changes in hepatic redox indices, further indicating that the present data do not support its designation as an optimal or recommended inclusion level. The increases in AST and LDL-C in FGS30 also contrast with the reductions in liver-related enzymes and lipid indices previously reported in grape-pomace-supplemented laying hens [24], further indicating that species, physiological stage, product composition, inclusion level, and basal-diet conditions may influence the metabolic response to grape by-products.

### 4.4. Carcass Traits and Organ Development

FGS30 increased carcass yield, semi-eviscerated yield, and eviscerated yield but did not increase carcass weight, semi-eviscerated weight, eviscerated weight, or absolute muscle weights. Because preslaughter live weight was numerically lower in FGS30, the higher relative carcass yields may partly reflect a denominator effect and do not necessarily indicate greater absolute meat production per squab. Therefore, the effect of FGS30 on carcass traits should be interpreted as a change in relative carcass proportions rather than an improvement in absolute meat yield.

Preslaughter live weight, carcass weight, semi-eviscerated weight, eviscerated weight, and thigh muscle weight were numerically higher in FGS20, but none of these differences was significant. The absolute and relative weights of the major organs also showed no significant treatment effects. These findings indicate a relatively stable final production profile in FGS20 but do not demonstrate a significant increase in absolute carcass output. FGS supplementation did not significantly affect the relative weights of the breast or thigh muscles. Together with the absence of significant differences in their absolute weights, these findings indicate that FGS did not markedly alter breast or thigh muscle deposition at 28 d of age.

### 4.5. Meat-Quality Changes and Application Limitations

FGS mainly altered squab muscle color and water-holding capacity without significantly affecting shear force. Breast muscle L* values were higher in FGS20 and FGS30, and selected a* and b* values also differed in FGS20, indicating altered meat color. Because consumer preference, sensory quality, and shelf life were not assessed, increases in L*, a*, or b* cannot be interpreted directly as improved commercial appearance. In the CIE color system, L* represents lightness, with higher values indicating lighter meat; a* represents the red–green axis, with higher positive values indicating greater redness; and b* represents the yellow–blue axis, with higher positive values indicating greater yellowness. In the present study, the higher breast muscle L* values in FGS20 and FGS30 indicate a lighter breast meat appearance, whereas the higher breast muscle b* value in FGS20 indicates greater yellowness. The increases in thigh muscle a* and b* indicate increased redness and yellowness, respectively. These findings demonstrate that FGS altered the instrumental color characteristics of squab meat.

Breast muscle press loss was significantly increased in FGS20 and FGS30, indicating reduced water-holding capacity, and thigh muscle press loss also increased linearly. From a practical perspective, reduced water-holding capacity may be unfavorable because greater water loss may reduce processing yield and affect the technological and sensory quality of meat. Therefore, although FGS20 showed relatively favorable responses in several production-related traits, the increased breast muscle press loss should be considered when evaluating its practical application. Because processing yield, sensory characteristics, and consumer acceptance were not directly assessed, these practical implications require further validation. Breast and thigh muscle shear force did not differ significantly, indicating that FGS did not alter instrumentally measured tenderness under the present conditions. The changes in meat color and water-holding capacity observed here are consistent with the variable meat-quality responses reported for grape-derived ingredients in poultry, which appear to depend on ingredient composition, inclusion rate, and measurement conditions [22,23,30]. Therefore, the increased L* values and press loss in the present study should be regarded as responses specific to the current experimental conditions rather than as general effects of grape by-products.

### 4.6. Integrated Evaluation and Directions for Further Validation

Taken together, the overall biomarker profile did not indicate a uniform improvement in physiological status. In FGS30, increased immunoglobulin concentrations occurred alongside higher TNF-α and MDA and reduced hepatic antioxidant enzyme activities, indicating immune modulation accompanied by greater inflammatory and oxidative burden rather than an unequivocal health benefit. Among the supplemented groups, fewer unfavorable serum changes were observed in FGS20 than in FGS30, but FGS20 did not provide a clear overall advantage over CON. Its numerical advantages in 28 d body weight, ADG from 21 to 28 d, and several absolute carcass weights were not statistically significant, whereas breast muscle press loss increased and hepatic redox status was not improved. Accordingly, FGS20 should not be interpreted as an optimal or recommended inclusion level on the basis of the present data.

Future studies should evaluate more closely spaced inclusion levels across the range of 10–20 kg FGS per 1000 kg basal diet and chemically analyze each of the four final mixed diets. Specific polyphenol constituents, including proanthocyanidins, as well as tannins, individual organic acids, and major fermentation metabolites should also be characterized. Full-period cumulative feed consumption per family cage and economic returns should be recorded, and hepatic histology, meat drip loss and cooking loss, sensory evaluation, and vaccine or pathogen-challenge models should be incorporated to verify production and health effects.

### 4.7. Study Limitations

This study was conducted at a single commercial pigeon farm using one batch of FGS. Because FGS was applied as an additional dietary supplement rather than as a replacement for a specific basal-diet ingredient, the experimental diets were not formulated to be either isonitrogenous or isoenergetic. Accordingly, the present findings should be interpreted as the overall responses to FGS supplementation under the conditions of this study. The chemical composition of the four final mixed diets was not analyzed separately, and specific polyphenol profiles, tannins, individual organic acids, and major fermentation metabolites in the product were not characterized. Feed consumption per family cage was measured continuously for 24 h at four ages and therefore cannot replace full-period cumulative feed intake or be used to calculate a conventional feed conversion ratio for squabs. Immune indices were based on static serum biomarkers; hepatic redox changes lacked histological verification; and the integrated evaluation of FGS20 was not based on a prespecified composite endpoint or weighted score. Thus, the present study identifies dose-related responses and suggests a range for further validation, but production recommendations and mechanistic extrapolations should be made cautiously.

## 5. Conclusions

Under the conditions of this study, FGS supplementation in breeding-pigeon diets produced dose-related effects on early squab growth, relative carcass yields, meat color, water-holding capacity, immune-related responses, and redox status. FGS30 increased immunoglobulin concentrations and relative carcass yields but reduced ADG from 14 to 21 and 14 to 28 d, increased TNF-α, AST, LDL-C, and serum and hepatic MDA, and decreased hepatic SOD and GSH-Px, suggesting that this inclusion level may exceed the appropriate range. FGS20 showed numerical advantages in 28 d body weight, ADG from 21 to 28 d, and several absolute carcass weights, but these differences were not statistically significant; breast muscle press loss increased, and hepatic redox status was not improved. Overall, FGS30 was associated with more unfavorable responses and may exceed the appropriate inclusion range. Based on the present data, none of the tested supplementation levels can be considered optimal or recommended, and the range of 10–20 kg FGS per 1000 kg basal diet requires further validation.

## Figures and Tables

**Table 1:** Analyzed chemical composition and selected physicochemical characteristics of fermented grape seed (FGS).

Item	Content
Dry matter (% as-fed)	94.17
Crude protein (% DM)	17.82
Ether extract (% DM)	16.80
Neutral detergent fiber (% DM)	62.70
Acid detergent fiber (% DM)	50.09
Ash (% DM)	8.99
Total phenols (mg GAE/g)	20.25
Total flavonoids (mg RE/g)	19.16
Total acids (g/kg, as lactic acid)	4.88
pH	4.20

Note: DM, dry matter; GAE, gallic acid equivalents; RE, rutin equivalents. Crude protein, ether extract, neutral detergent fiber, acid detergent fiber, and ash are expressed on a dry-matter basis.

**Table 2:** Ingredient composition and calculated nutrient content of the basal diet and estimated proximate composition of the experimental diets.

Ingredient Composition and Calculated Nutrient Content of the Basal Diet				Estimated Proximate Composition of the Experimental Diets (% of DM)				
Item	Content (kg/1000 kg)	Item	Content (kg/1000 kg)	Item	CON	FGS10	FGS20	FGS30
Corn	621.50	Peas	150.00	Crude protein	17.06	17.07	17.08	17.08
Wheat middlings	24.00	Sodium bicarbonate	4.00	Ether extract	4.83	4.95	5.07	5.19
Salt	4.00	Yeast powder	20.00	Neutral detergent fiber	16.26	16.73	17.19	17.64
Soybean meal	140.00	Dicalcium phosphate	6.00	Acid detergent fiber	5.35	5.80	6.25	6.68
Mycotoxin binder	0.50	DL-Methionine	1.60	Ash	10.26	10.25	10.23	10.22
L-Lysine	2.40	Limestone	8.00					
Soybean oil	10.00	Compound premix	8.00					
Total	1000.00							
Calculated nutrient content of the basal diet								
Metabolizable energy (MJ/kg)	11.89	Crude protein (%)	15.79					
Calcium (%)	0.54	Total phosphorus (%)	0.32					
Lysine (%)	0.91	Methionine (%)	0.39					

Notes: The premix was included in the complete diet at 8 kg/t, corresponding to an inclusion rate of 0.80%. Per kilogram of complete diet, the premix supplied 12,000 IU of vitamin A, 40,000 IU of vitamin D_3_, 377.78 mg of vitamin E, 40.89 mg of vitamin K_3_, 71.11 mg of vitamin B_2_, 51.11 mg of vitamin B_6_, 466.67 mg of nicotinamide, 231.11 mg of calcium D-pantothenate, 0.89 mg of D-biotin, 8.89 mg of folic acid, 151.11 mg of Cu, 755.56 mg of Fe, 755.56 mg of Mn, and 622.22 mg of Zn.

**Table 3:** Effects of dietary supplementation with fermented grape seed (FGS) on 24 h feed consumption per family cage and the growth performance of squabs.

Item	CON	FGS10	FGS20	FGS30	Pooled SEM	*p*-Value		
						Treatment	Linear	Quadratic
**Body weight (g/bird)**								
14 d	337.19 ^b^	365.28 ^a^	369.81 ^a^	384.47 ^a^	9.80	0.012	0.002	0.497
21 d	431.83	443.83	448.33	440.58	9.77	0.678	0.485	0.318
28 d	459.78	468.22	478.14	464.58	9.08	0.537	0.552	0.232
**Average daily gain (g/bird/d)**								
14–21 d	13.52 ^a^	11.22 ^a^	11.22 ^a^	8.02 ^b^	0.97	0.003	<0.001	0.643
21–28 d	3.99	3.48	4.26	3.43	1.03	0.928	0.844	0.877
14–28 d	8.76 ^a^	7.35 ^ab^	7.74 ^a^	5.72 ^b^	0.66	0.020	0.005	0.646
**24 h feed consumption per family cage (g/cage/24 h)**								
7 d	155.00	157.25	144.25	159.58	6.20	0.323	0.979	0.297
14 d	210.83	219.08	210.83	226.33	8.49	0.515	0.319	0.671
21 d	245.08	243.33	234.75	239.00	9.52	0.870	0.532	0.754
28 d	179.25	181.75	179.67	183.58	7.52	0.975	0.747	0.925
**Repeated-measures model results**								
Response variable						Treatment *p*	Age *p*	Treatment × Age *p*
Body weight						0.239	<0.001	0.013
24 h feed consumption per family cage						0.504	<0.001	0.964

Notes: CON, FGS10, FGS20, and FGS30 denote the addition of 0, 10, 20, and 30 kg of FGS per 1000 kg basal diet, respectively. Each treatment included 12 replicates, with cage as the experimental unit. Body weight and ADG are the means of three squabs per cage. Feed consumption per family cage represents the amount of feed consumed during a continuous 24 h measurement at the corresponding age and is not cumulative intake. Data are presented as means and pooled SEM. Treatment *p*-values are from one-way analysis of variance at each age or interval; linear and quadratic *p*-values are from orthogonal polynomial contrasts. The overall treatment, age, and treatment × age effects from the repeated-measures models are presented in the lower section of Table 3. Within a row, means with different lowercase superscript letters differ significantly (*p* < 0.05; Duncan’s test).

**Table 4:** Effects of graded FGS supplementation on serum and hepatic redox status in 28-day-old squabs.

Variable	CON	FGS10	FGS20	FGS30	Pooled SEM	Treatment *p*	Linear *p*	Quadratic *p*
**Serum redox indices**								
T-SOD (U/mL)	153.07	152.06	154.07	168.73	7.06	0.306	0.128	0.274
MDA (nmol/mL)	3.63 ^b^	3.68 ^b^	4.37 ^a^^b^	5.41 ^a^	0.33	0.001	<0.001	0.146
GSH-Px (U/mL)	1376.74 ^b^	1673.26 ^a^^b^	1498.84 ^b^	1970.93 ^a^	107.15	0.002	0.002	0.417
CAT (U/mL)	0.534	0.517	0.511	0.432	0.046	0.425	0.141	0.507
T-AOC (mmol/L)	0.428	0.396	0.404	0.443	0.017	0.202	0.494	0.044
**Hepatic redox indices**								
SOD (U/mg protein)	328.77 ^a^	292.94 ^b^	305.59 ^a^^b^	286.03 ^b^	6.70	<0.001	<0.001	0.231
MDA (nmol/mg protein)	0.524 ^b^	0.691 ^a^	0.662 ^a^	0.697 ^a^	0.035	0.003	0.003	0.061
Hepatic GSH-Px (U/mg protein)	79.54 ^a^	66.80 ^b^	67.52 ^b^	63.04 ^b^	1.98	<0.001	<0.001	0.043
Hepatic T-AOC (mmol/g protein)	0.0228	0.0192	0.0181	0.0198	0.0012	0.061	0.080	0.035

Notes: *n* = 12 per group, with cage as the experimental unit. Data are presented as means and pooled SEM. T-SOD, total superoxide dismutase; SOD, superoxide dismutase; MDA, malondialdehyde; GSH-Px, glutathione peroxidase; CAT, catalase; T-AOC, total antioxidant capacity. Within a row, means with different lowercase superscript letters differ significantly (*p* < 0.05). Linear and quadratic *p*-values were obtained from prespecified orthogonal polynomial contrasts.

**Table 5:** Effects of graded FGS supplementation on serum immunoglobulins and inflammatory cytokines in squabs.

Variable	CON	FGS10	FGS20	FGS30	Pooled SEM	Treatment *p*	Linear *p*	Quadratic *p*
IgA (mg/mL)	2.13 ^b^	2.34 ^b^	2.59 ^b^	3.37 ^a^	0.15	<0.001	<0.001	0.063
IgG (mg/mL)	5.03 ^b^	6.05 ^ab^	5.51 ^b^	7.05 ^a^	0.32	<0.001	<0.001	0.412
IgM (mg/mL)	1.61 ^b^	1.87 ^b^	1.86 ^b^	2.46 ^a^	0.10	<0.001	<0.001	0.115
IL-1β (ng/L)	17.74	19.29	18.41	23.84	1.51	0.122	—	—
IL-6 (ng/L)	70.98	70.26	83.62	84.43	4.69	0.055	0.014	0.872
IL-10 (ng/L)	76.92 ^b^	84.65 ^b^	82.72 ^b^	101.30 ^a^	3.55	<0.001	<0.001	0.134
TNF-α (ng/L)	66.79 ^b^	72.29 ^b^	72.45 ^b^	93.34 ^a^	4.14	<0.001	<0.001	0.070

Notes: *n* = 12 per group, with cage as the experimental unit. IgA, immunoglobulin A; IgG, immunoglobulin G; IgM, immunoglobulin M; IL, interleukin; TNF-α, tumor necrosis factor-α. Within a row, means with different lowercase superscript letters differ significantly (*p* < 0.05; Duncan’s test). Variance was heterogeneous for IL-1β, and its treatment *p*-value was obtained using Welch’s test; the remaining indices were analyzed by one-way analysis of variance. Linear and quadratic *p*-values correspond to the respective orthogonal polynomial contrasts. CON, FGS10, FGS20, and FGS30 denote the addition of 0, 10, 20, and 30 kg FGS, respectively, per 1000 kg basal diet.

**Table 6:** Effects of graded FGS supplementation on serum biochemical and lipid-metabolism parameters in 28-day-old squabs.

Parameter	CON	FGS10	FGS20	FGS30	Pooled SEM	Treatment *p*	Linear *p*	Quadratic *p*
TP, g/L	19.72	21.87	19.30	20.08	0.95	0.247	0.725	0.475
ALB, g/L	11.41	12.75	11.01	12.33	0.49	0.058	0.644	0.980
ALT, U/L	3.01	2.55	2.27	2.70	0.26	0.257	0.309	0.094
AST, U/L	5.72 ^b^	5.94 ^b^	5.92 ^b^	8.27 ^a^	0.52	0.003	0.002	0.048
TG, mmol/L	1.009	1.009	0.953	0.911	0.080	0.793 †	—	—
TC, mmol/L	9.37	9.60	9.41	10.23	0.31	0.193	0.094	0.341
HDL-C, mmol/L	3.14	3.45	3.18	3.59	0.16	0.176	0.153	0.751
LDL-C, mmol/L	3.64 ^b^	4.38 ^a^^b^	4.21 ^a^^b^	5.04 ^a^	0.29	0.013	0.003	0.876

Note: Each treatment included 12 replicates, with cage as the experimental unit. Data are presented as means with the pooled standard error of the mean (pooled SEM). Within a row, means with different lowercase superscript letters differ significantly (*p* < 0.05; Duncan’s multiple-range test). † The TG data did not meet the assumption of homogeneity of variance; therefore, the treatment *p*-value was obtained using Welch’s test.

**Table 7:** Effects of graded FGS supplementation on carcass traits of 28-day-old squabs.

Parameter	CON	FGS10	FGS20	FGS30	Pooled SEM	Treatment *p*	Linear *p*	Quadratic *p*
Preslaughter live weight (g)	440.08	446.42	450.83	423.17	9.10	0.165	0.261	0.069
Carcass weight (g)	367.79	371.85	374.68	363.56	7.27	0.723	0.764	0.302
Carcass yield (%)	83.61 ^b^	83.37 ^b^	83.12 ^b^	85.94 ^a^	0.52	0.001	0.006	0.005
Semi-eviscerated weight (g)	344.72	348.34	353.29	343.10	7.35	0.769	0.998	0.352
Semi-eviscerated yield (%)	78.35 ^b^	78.09 ^b^	78.38 ^b^	81.09 ^a^	0.74	0.020	0.014	0.052
Eviscerated weight (g)	313.17	318.06	322.73	313.43	7.12	0.751	0.865	0.324
Eviscerated yield (%)	71.15 ^b^	71.30 ^b^	71.61 ^b^	74.08 ^a^	0.79	0.040	0.014	0.150
Breast muscle weight (g)	35.49	36.72	36.71	36.57	1.60	0.939	0.657	0.671
Thigh muscle weight (g)	9.36	9.33	10.62	9.66	0.41	0.105	0.240	0.263
Relative breast muscle weight (%)	11.34	11.50	11.23	11.61	0.37	0.814 †	0.739	0.768
Relative thigh muscle weight (%)	2.98	2.93	3.26	3.07	0.10	0.101	0.174	0.466
Abdominal fat weight (g)	2.545	2.605	3.132	2.681	0.357	0.646	0.562	0.478
Abdominal fat percentage (%)	0.571	0.584	0.692	0.627	0.078	0.687	0.430	0.618

Note: Each treatment included 12 replicates, with cage as the experimental unit. Data are presented as means with the pooled standard error of the mean (pooled SEM). Except for variables marked with †, treatment *p*-values were obtained by one-way analysis of variance; the linear and quadratic *p*-values were obtained from linear and quadratic orthogonal polynomial contrasts, respectively. Within a row, means with different lowercase superscript letters differ significantly (*p* < 0.05; Duncan’s multiple-range test). † The relative breast muscle weight data did not meet the assumption of homogeneity of variance; therefore, the treatment *p*-value was obtained using Welch’s test.

**Table 8:** Effects of graded FGS supplementation on breast and thigh muscle quality in 28-day-old squabs.

Parameter	CON	FGS10	FGS20	FGS30	Pooled SEM	Treatment *p*	Linear *p*	Quadratic *p*
Breast muscle pH	6.08	6.04	5.94	5.85	0.072	0.112 †	0.013	0.780
Thigh muscle pH	6.22	6.25	6.27	6.29	0.063	0.747 †	0.310	0.993
Breast muscle L*	31.72 ^b^	30.81 ^b^	36.35 ^a^	35.31 ^a^	0.98	<0.001 †	<0.001	0.946
Breast muscle a*	17.34	17.39	19.36	18.41	0.77	0.210	0.137	0.518
Breast muscle b*	12.22 ^b^	12.14 ^b^	15.46 ^a^	14.20 ^ab^	0.80	0.013	0.014	0.466
Thigh muscle L*	36.84	37.63	39.13	39.35	1.44	0.558	0.168	0.846
Thigh muscle a*	12.52 ^c^	14.10 ^bc^	17.06 ^a^	15.38 ^ab^	0.85	0.004	0.004	0.062
Thigh muscle b*	12.32 ^c^	15.28 ^b^	17.28 ^a^	16.03 ^ab^	0.59	<0.001	<0.001	<0.001
Breast muscle press loss (%)	10.26 ^b^	9.53 ^b^	21.61 ^a^	19.15 ^a^	1.92	<0.001	<0.001	0.655
Thigh muscle press loss (%)	8.36	10.77	10.91	18.76	2.24	0.068 †	0.019	0.238
Breast muscle shear force (N)	25.48	25.92	29.68	26.86	1.76	0.337	0.319	0.358
Thigh muscle shear force (N)	22.72	21.90	25.35	19.91	1.80	0.213	0.540	0.208

Note: Each treatment included 12 replicates, with cage as the experimental unit. Data are presented as means with the pooled standard error of the mean (pooled SEM). For variables marked with †, the assumption of homogeneity of variance was not met, and the treatment *p*-value was obtained using Welch’s test. Pairwise comparisons for breast muscle L* were performed using the Games–Howell test, whereas Duncan’s multiple-range test was used for the other significant variables. The linear and quadratic *p*-values correspond to the respective orthogonal polynomial contrasts. Within a row, means with different lowercase superscript letters differ significantly (*p* < 0.05).

## Data Availability

The raw data supporting the conclusions of this study are available from the corresponding author upon reasonable request.

## Supplementary Materials

The following supporting information can be downloaded at: https://www.mdpi.com/article/10.3390/ani16172692/s1. Supplementary Table S1. Complete body measurements of squabs from 14 to 28 d of age. Supplementary Table S2. Organ weights and organ indices of squabs.
