# Supplementary material for: Effects of Fermented Grape Seed in Breeding-Pigeon Diets on Growth Performance, Immune Responses, and Oxidative Status of Offspring Squabs
Source: Animals (Basel). 2026 Aug 29;16(17):2692. doi: 10.3390/ani16172692 (PMC13564300; doi:10.3390/ani16172692)
Supplement: Supplementary file 1 [file animals-16-02692-s001.zip › animals-4515212-supplementary.pdf]

Supplementary Materials

The Supplementary Materials contain the complete body-measurement and organ data that are not fully presented in the main text, thereby avoiding duplication of the main tables.

Supplementary Table S1. Complete body measurements of squabs from 14 to 28 d of age.

Variable	CON	FGS10	FGS20	FGS30	Pooled SEM	Treatment P	Linear P	Quadratic P
14 d body oblique length	8.93[b]	9.24[ab]	9.66[a]	9.53[a]	0.13	<0.001	<0.001	0.088
14 d keel length	5.04[b]	5.27[ab]	5.29[ab]	5.56[a]	0.11	0.013	0.002	0.888
14 d shank circumference	2.11	2.17	2.18	2.17	0.02	0.095	0.044	0.141
14 d shank length	27.81	28.28	29.22	29.41	0.45	0.048	0.007	0.757
14 d chest width	46.64[b]	49.51[ab]	51.55[a]	50.61[a]	0.93	0.004	0.002	0.047
14 d chest depth	54.49	55.94	55.69	56.80	0.85	0.293	0.083	0.843
21 d body oblique length	10.91	11.19	11.08	11.01	0.12	0.427	0.735	0.154
21 d keel length	6.91	7.23	6.88	7.25	0.12	0.041	0.211	0.811
21 d shank circumference	2.18	2.17	2.21	2.18	0.02	0.273	0.550	0.533
21 d shank length	31.88	32.26	31.48	32.18	0.35	0.386	0.934	0.648
21 d chest width	60.61	61.43	61.29	61.07	0.72	0.860	0.697	0.472
21 d chest depth	61.74	62.41	61.89	64.27	0.78	0.096	0.048	0.279
28 d body oblique length	10.91	11.20	11.45	11.38	0.16	0.081	0.022	0.247
28 d keel length	7.63	7.97	7.85	7.94	0.15	0.378	0.237	0.400
28 d shank circumference	2.16[b]	2.22[ab]	2.22[ab]	2.24[a]	0.02	0.030	0.006	0.361
28 d shank length	31.82	31.93	31.87	32.33	0.32	0.671	0.316	0.589
28 d chest width	60.59[b]	62.09[ab]	64.62[a]	62.89[ab]	0.73	0.003	0.006	0.032
28 d chest depth	66.76	66.73	67.14	67.33	0.60	0.868	0.432	0.856

Notes: n = 12 cages per group, with cage as the experimental unit. CON, FGS10, FGS20, and FGS30 denote the addition of 0, 10, 20, and 30 kg FGS, respectively, per 1000 kg basal diet. Body oblique length, keel length, and shank circumference are expressed in cm; shank length, chest width, and chest depth are expressed in mm.

Supplementary Table S2. Organ weights and organ indices of squabs.

Parameter	CON	FGS10	FGS20	FGS30	Pooled SEM	Treatment P	Linear P	Quadratic P
Heart weight (g)	4.626	5.059	4.932	4.903	0.194	0.455	0.420	0.240
Liver weight (g)	11.839	10.720	10.063	9.407	0.784	0.171	0.028	0.769
Spleen weight (g)	0.449	0.443	0.401	0.388	0.050	0.776	0.318	0.947
Kidney weight (g)	3.012	3.087	3.220	3.037	0.222	0.913	0.835	0.564
Proventriculus weight (g)	1.109	1.107	1.146	1.139	0.041	0.867	0.485	0.960
Gizzard weight (g)	6.872	6.690	7.213	6.768	0.346	0.723	0.891	0.706
Bursa of Fabricius weight (g)	0.708	0.788	0.756	0.707	0.066	0.781	0.906	0.331
Heart index (%)	1.055	1.133	1.094	1.156	0.039	0.280	0.134	0.841
Liver index (%)	2.708	2.409	2.234	2.225	0.181	0.213	0.051	0.427
Spleen index (%)	0.103	0.099	0.088	0.092	0.012	0.794	0.383	0.726
Kidney index (%)	0.694	0.692	0.716	0.717	0.052	0.976	0.690	0.964
Proventriculus index (%)	0.254	0.248	0.255	0.269	0.010	0.513	0.245	0.341
Gizzard index (%)	1.564	1.500	1.593	1.606	0.072	0.733	0.502	0.597

Parameter	CON	FGS10	FGS20	FGS30	Pooled SEM	Treatment P	Linear P	Quadratic P
Bursa of Fabricius index (%)	0.160	0.176	0.166	0.167	0.013	0.865	0.835	0.562

Note: Each treatment included 12 replicates, with cage as the experimental unit. Organ indices were calculated as the percentage of organ weight relative to preslaughter live weight. Data are presented as means with the pooled standard error of the mean (pooled SEM).
